# Therapeutic Effects of Alkaloids on Influenza: A Systematic Review and Meta-Analysis of Preclinical Studies

**DOI:** 10.3390/ijms26051823

**Published:** 2025-02-20

**Authors:** Zhaoyuan Gong, Mingzhi Hu, Guozhen Zhao, Ning Liang, Haili Zhang, Huizhen Li, Qianzi Che, Jing Guo, Tian Song, Yanping Wang, Nannan Shi, Bin Liu

**Affiliations:** Institute of Basic Research in Clinical Medicine, China Academy of Chinese Medical Sciences, Beijing 100700, China; gzy1206305166@126.com (Z.G.); hmzz930@163.com (M.H.); 13811582123@163.com (G.Z.); liangning229@163.com (N.L.); hailizhang0127@126.com (H.Z.); heyitsokj@126.com (H.L.); cheqianzi123@126.com (Q.C.); 18514573788@163.com (J.G.); songnait@126.com (T.S.); wangyanping4816@163.com (Y.W.)

**Keywords:** alkaloid, influenza, meta-analysis, systematic review

## Abstract

Experimental evidence suggests that alkaloids have anti-influenza and anti-inflammatory effects. However, the risk of translating existing evidence into clinical practice is relatively high. We conducted a systematic review and meta-analysis of animal studies to evaluate the therapeutic effects of alkaloids in treating influenza, providing valuable references for future studies. Seven electronic databases were searched until October 2024 for relevant studies. The Review Manager 5.2 software was utilized to perform the meta-analysis. Our study was registered within the International Prospective Register of Systematic Reviews (PROSPERO) as number CRD42024607535. Alkaloids are significantly correlated with viral titers, pulmonary inflammation scores, survival rates, lung indices, and body weight. However, alkaloid therapy is not effective in reducing the levels of tumor necrosis factor-α (TNF-α) and interleukin-6 (IL-6). In addition, the therapeutic effects of alkaloids may be related to the inhibition of the Toll-like receptor 4 or 7/Nuclear factor (NF)-κB signaling pathway, NACHT, LRR, and PYD domains-containing protein 3 (NLRP3) inflammasome pathway, and the Antiviral innate immune response receptor RIG-I (RIG-I) pathway. Alkaloids are potential candidates for the prevention and treatment of influenza. However, extensive preclinical studies and clinical studies are needed to confirm the anti-influenza and anti-inflammatory properties of alkaloids.

## 1. Introduction

Influenza is a contagious disease that affects people worldwide, causing recurrent respiratory diseases in humans [1]. It can develop in various ways, from asymptomatic infections or mild upper respiratory infections tract to serious illnesses with high fever, chills, muscle pain, and pneumonia, and eventually develop into acute respiratory distress syndrome (ARDS) and death from respiratory failure [2]. According to the World Health Organization, approximately one billion people are infected, and up to 500,000 people die from influenza every year [3]. In addition, antigenic drift and antigenic transfer can lead to periodic epidemics of influenza viruses. Influenza poses a global health, medical, and economic burden [4]. The influenza strains currently circulating in humans include influenza A (H1N1) pdm09, influenza A (H3N2), and both influenza B viruses [5]. There are multiple drug treatment options that can inhibit specific sequences of pathological processes of influenza. However, only two classes of anti-influenza drugs have been approved globally. Moreover, the sustained mutations and resistance of the virus mean that existing drugs and vaccines can only provide limited help in controlling influenza [6]. As the effectiveness of vaccines and drugs decreases, developing more effective, safe, and affordable treatment options is an immediate challenge [7].

According to reports, the host’s inflammatory response to the influenza virus is more related to lung injury caused by the influenza virus [8,9]. The airway epithelial cells lining the respiratory mucosa are the main targets of influenza infection. Influenza virus antigens are recognized by presenting antigen cells and pattern recognition receptors and can up-regulate several corresponding down-stream molecules, including interleukin-6 (IL-6), IL-1β, and tumor necrosis factor-alpha (TNF-α) [10]. If excessive production of pro-inflammatory cytokines leads to an aggressive pro-inflammatory response and insufficient control of the anti-inflammatory response, this series of events is called a cytokine storm, which can lead to major immunopathology and serious disease consequences [11,12]. In addition, research has shown that influenza viruses can hijack a series of intracellular signaling cascades for their own benefit, such as Toll-like receptor (TLR), Antiviral innate immune response receptor RIG-I (RIG-I), Protein kinase C (PKC)/Prokineticin receptor (PKR), Phosphatidylinositol 3-kinase (PI3K)/serine/threonine-protein kinase (Akt), Mitogen-activated protein kinase (MAPK), and Nuclear factor NF-kappa-B (NF-κB) signaling pathways [13]. There are further opportunities to improve the investigation of the pathogenesis of influenza. Hence, intensive research into the pathogenesis of influenza and the exploration of effective therapeutic agents are crucial for clinical management.

Alkaloids are organic metabolites derived from microorganisms, plants, and animals, which have attracted tremendous attention due to their wide clinical applications and have shown promising therapeutic benefits in antiviral, cough suppressant, analgesic, and anti-inflammatory [14,15,16,17]. It is usually a nitrogenous small molecule derived from plants and exhibits basicity due to the presence of nitrogen [18]. Both in vitro and in vivo studies have shown that alkaloids and their derivatives have strong anti-influenza activity, which can effectively reduce lung injury and improve immune function [19]. However, these findings in individual animal experiments are often influenced by multiple factors, such as small sample sizes, influenza animal models, and intervention duration. There are limitations in drawing reliable conclusions about the anti-influenza or anti-inflammatory properties of alkaloids in the treatment of influenza. In addition, methodological quality and publication bias of animal experiments are still unclear, which may exaggerate the efficacy of alkaloids [20].

Systematic analysis based on animal data provides valuable insights into the reliability of animal research, improves the accuracy of estimation results, and facilitates the transition to human clinical trials [21,22]. A review based on animal data can improve the planning of trials, increase the success rate of future clinical trials, and help determine which ones are valuable in further research. Therefore, this study aims to conduct a systematic review and meta-analysis of preclinical evidence on the treatment of influenza with alkaloid drugs in animal models to analyze the efficacy and potential mechanisms of alkaloid therapy for influenza. Our findings provide a scientific basis for using alkaloids to treat influenza and offer suggestions for future animal studies and clinical trials.

## 2. Materials and Methods

The systematic review and meta-analysis in this study were conducted according to the Cochrane Handbook for Systematic Reviews of Interventions and adhered to the Preferred Reporting Items for Systematic Reviews and Meta-Analysis guidelines [23]. The protocol is available on the PROSPERO website under registration number CRD42024607535, utilizing SYRCLE’s tool for animal studies [24].

### 2.1. Search Strategies

Two authors (Z. Gong and M. Hu) independently conducted searches of electronic bibliographic databases, including PubMed, Embase, Web of Science, China National Knowledge Internet (CNKI), VIP Information Chinese Periodical Service Platform (VIP), China Biology Medicine Disc (CBM), and Wanfang Data Knowledge Service Platform (Wanfang) to identify relevant animal studies from database inception until October 2024. We used medical subject headings (MeSH) and free-text terms in our search, tailored for each database without language or publication year restrictions. The MeSH terms were as follows: (“Influenza, Human” OR “Human Influenzas” OR “Influenzas, Human” OR “Influenza” OR “Influenzas” OR “Human Flu” OR “Flu, Human” OR “Human Influenza” OR “Influenza in Humans” OR “Influenza in Human” OR “Grippe”) AND (“Alkaloids” OR “Alkaloid” OR “Plant Alkaloids” OR “Alkaloids, Plant” OR “Plant Alkaloid” OR “Alkaloid, Plant”). 

### 2.2. Inclusion and Exclusion Criteria

Inclusion criteria were as follows: (1) Participants: influenza model animals; (2) Intervention: alkaloid monomer compounds (pure alkaloids) and subclasses of alkaloids with the duration of administration and dose clarified; (3) Comparison: control group was an influenza group with no treatment; (4) Outcomes: viral titer, pulmonary inflammation score, and survival rate were the primary outcomes, lung index, IL-6, TNF-α, and body weight were the secondary outcomes; (5) Study design: controlled studies with a separate control group; (6) Language: English.

Exclusion criteria were as follows: (1) animals with co-morbidity, in vitro studies, clinical trials, reviews, and case reports; (2) experimental group administered a mixture of alkaloid extracts or without a precise dose and duration of administration; (3) control group lacked an accurate influenza virus induction dose; (4) non-influenza models; (5) no relevant outcomes; (6) duplicate publication; (7) studies without full text; (8) the language is non-Chinese or non-English.

### 2.3. Study Screening and Data Extraction

The retrieved literature was managed using NoteExpress (X9 version). After duplication, two authors (Z. Gong and M. Hu) independently screened based on the title and abstract, then screened the full text of the potentially eligible articles for final determination according to the inclusion and exclusion criteria [25]. The disagreements about whether a study should be included were resolved through discussion with the corresponding author (B. Liu).

Two reviewers independently extracted basic information from the included studies and recorded details by Excel 2019 software, including (1) basic information: first author’s surname and year of publication; (2) intervention (alkaloid subclass, administration route, animal species, treatment duration); (3) information on subjects: sample size, weight, species, and induction method for animal models of influenza in the experimental group and control group; (4) outcome measures: viral titer, pulmonary inflammation score, survival rate, lung index, IL-1β, IL-6, TNF-α, and body weight. When multiple alkaloid intervention doses were tested in the study, data from the group receiving the lowest effective dose were recorded. All the outcome measures were continuous data, so we extracted the mean, standard deviation (SD), and number of participants (*n*). When presenting results at multiple time points, we extracted data from the last time point. If the outcome measures were presented only in graphical form, we contacted the corresponding authors of the relevant studies to obtain the raw data. When it could not be implemented, we used the GetData Graph Digitizer (version 2.26) to quantify the results. Any disagreements between reviewers regarding data extraction were resolved through discussions with the corresponding author.

### 2.4. Quality and Risk Assessment

The SYstematic Review Center for Laboratory animal Experimentation (SYRCLE) risk-of-bias assessment tool was utilized to independently evaluate the methodological quality of included studies by two investigators. A ten-item checklist of evaluation criteria was as follows: (1) random sequence generation (selection bias); (2) balance of baseline characteristics (selection bias); (3) investigator and participants were unaware of intervention subgroups (selection bias); (4) random housing and (5) researcher blinding to evaluate performance bias; (6) random outcome assessment (detection bias); (7) blinding of the outcome measurer (detection bias); (8) incomplete outcome data, to evaluate attrition bias; (9) selective reporting, to evaluate reporting bias; (10) other issues that could lead to bias. We use “Yes” to indicate low risk, “No” to indicate high risk, and “Unclear” to indicate insufficient information to accurately assess bias risk [24].

### 2.5. Statistical Analysis

The Review Manager 5.2 software was utilized to perform the meta-analysis. All outcome indicators were continuous variables, so we expressed comparisons of overall effect size by standardized mean difference (SMD) and 95% confidence intervals (CI). When *p* < 0.05, it is considered statistically significant. A random-effects model was performed to calculate the pooled results. The heterogeneity between studies was evaluated using I^2^ statistics and a chi-squared test, where I^2^ > 50% indicated significant heterogeneity. We conducted a subgroup analysis based on the following variables to investigate potential sources of heterogeneity: influenza virus (A/Puerto Rico/8/34 [H1N1] (PR/8); A/FM1/1/47 [H1N1] (FM/1)). In addition, the impact of other potential factors on heterogeneity during the study was also explored, and variables with *p* < 0.05 were considered as sources of heterogeneity. When significant heterogeneity continued to exist and could not be resolved, we employed a random-effects model.

## 3. Results

### 3.1. Study Identification and Screening

**Figure 1:**
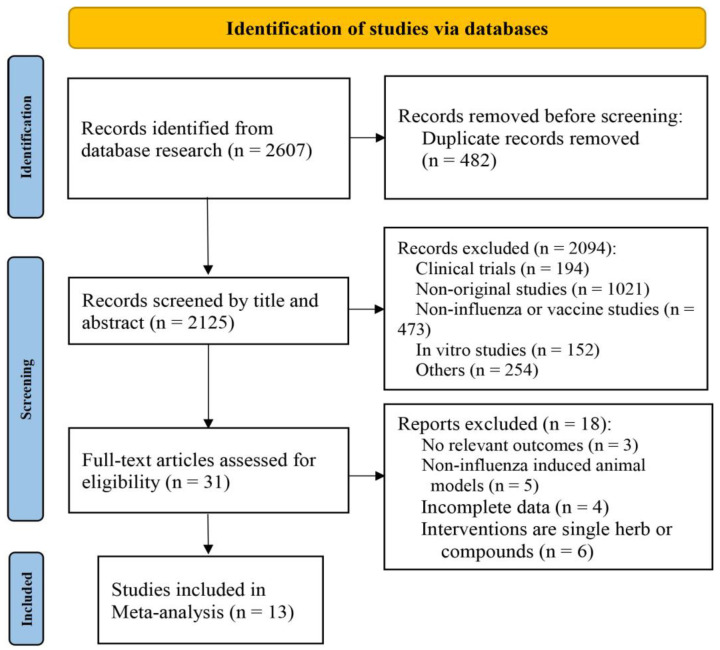
Flowchart of literature-screening process.

The systematic search collected a total of 2607 articles, including PubMed (387), Embase (1574), Web of Science (351), CNKI (46), CBM (130), Wanfang (99), and VIP (20). We adjusted for duplicates, and 2165 articles remained. Then, based on detailed reviews of their titles and abstracts, 2138 publications were excluded because of the following reasons: (1) clinical trials; (2) non-original studies; (3) non-influenza studies; (4) in vitro studies; (5) others. After a full-text review of 31 articles, 13 eligible studies were included in this systematic review and meta-analysis. The process of study selection is shown in Figure 1.

### 3.2. Characteristics of Search Results

A total of 420 influenza model animals (210 in the experimental group and 210 in the model group) and eight alkaloids were included in the 13 qualified studies. Among them, 12 studies used mice (92.3%), and 1 used rats (7.7%). The rat study used Lewis rats. In mice studies, seven used BALB/c mice, one used Kunming mice, one used ICR mice, three used C57BL/6 mice, and one study did not report the mouse strain. The weight of rats was reported in all studies, and the weight of mice ranged from 12 to 22 g. Three studies did not report the weight of the animals. All influenza animal models were induced by the influenza virus.

**Figure 2:**
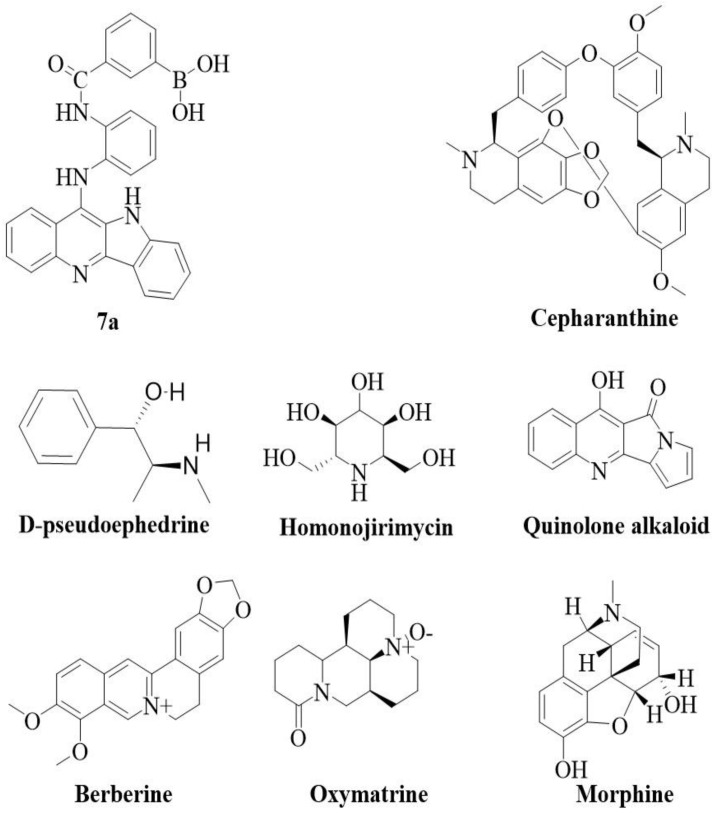
Structure of alkaloids included in the study.

The dosage range of alkaloids is 1 to 120 mg/kg/day. The routes of administration include oral gavage, intraperitoneal injection, and subcutaneous implant. The duration of administration ranged from 4 to 8 days. Control groups mainly included dimethyl sulfoxide (DMSO), saline, water, placebo, and phosphate buffer saline (PBS). Six studies (46.2%) used saline as the control group, three studies (23.1%) selected placebo, one study (7.7%) used DMSO, and three studies (23.1%) selected PBS. The characteristics of the 13 eligible studies are summarized in Table 1. The alkaloid structures included in the study are shown in Figure 2.

### 3.3. Quality of Qualified Studies

**Figure 3:**
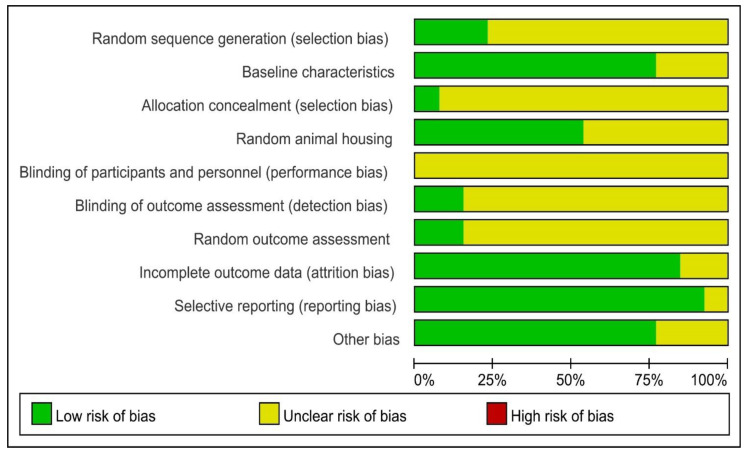
Risk of bias. Assessment of literature quality results obtained through a risk of bias by SYRCLE based on Cochrane tools.

The quality of all eligible studies was assessed using SYRCLE’s Risk of Bias tool (Figure 3). Random sequence generation and allocation concealment showed moderate to high proportions of unclear risk, with 10 and 12 studies, respectively, categorized as unclear. This suggests that randomization processes and allocation concealment were not consistently reported, raising potential concerns about selection bias.

In contrast, baseline characteristics were well-reported, with 10 studies indicating a low risk of bias, ensuring comparability between groups. Blinding of participants and personnel was associated with 13 unclear risk assessments, highlighting a significant gap in reporting or implementing blinding procedures. This may introduce potential performance bias affecting study outcomes. Blinding of outcome assessment revealed a notable degree of uncertainty, with 11 studies categorized as unclear risk. This indicates that most studies lacked sufficient information to confirm if outcome assessors were blinded, potentially introducing bias detection. Only a small proportion of studies (two studies) were classified as having a low risk in this domain. However, 11 studies were categorized as unclear for random outcome assessment, suggesting inconsistent reporting in some studies. Incomplete outcome data revealed a favorable profile, with 11 studies assessed as having a low risk of bias. This indicates that most studies effectively managed missing data, minimizing attrition bias. Selective reporting was predominantly classified as low risk (12 studies), reflecting comprehensive reporting of study outcomes across most eligible studies. Other potential biases were generally well-addressed, with 10 studies indicating a low risk. However, three studies were still classified as unclear, suggesting the possibility of unmeasured confounding factors.

### 3.4. Effect of Alkaloids on Survival Rate

**Figure 4:**
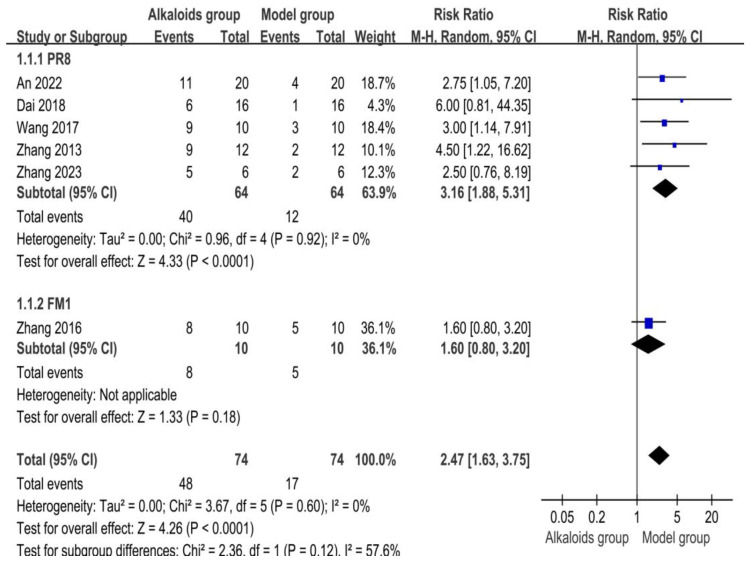
Effects of alkaloids on survival rate. The effects of alkaloids on survival rate compared to the model group were shown through a forest plot (effect size and 95% confidence interval). Black diamonds represented the pooled effect size. Blue squares represented the risk ratio, of each individual study, with the size of the square reflecting the weight of the study. The references cited in 1.1.1 PR8 were [27,28,30,32,33]; The reference cited in 1.1.2 FM1 was [38].

The effects of alkaloids on survival rate are shown in Figure 4. Five studies were included in the PR8 subgroup. The pooled risk ratio (RR) was 3.16 [95% CI: 1.88, 5.31], showing a significant increase in survival rate for the experimental group (Z = 4.33, *p* < 0.0001). No heterogeneity was detected (I^2^ = 0%, *p* = 0.92). Zhang et al. (2016) was the sole study in the FM1 subgroup. The RR was 1.60 [95% CI: 0.80, 3.20], suggesting no statistically significant improvement in survival (Z = 1.33, *p* = 0.18). Heterogeneity was not applicable. Combining all studies, the overall RR was 2.47 [95% CI: 1.63, 3.75], confirming a significant advantage for the experimental group (Z = 4.26, *p* < 0.0001). Subgroup analysis showed no substantial differences (Chi^2^ = 2.36, df = 1, *p* = 0.12; I^2^ = 57.6%).

### 3.5. Effect of Alkaloids on Viral Titer

**Figure 5:**
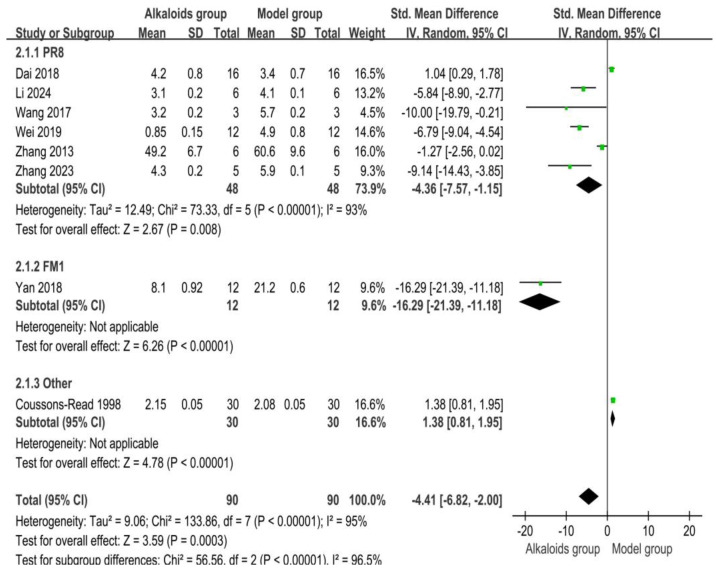
Effects of alkaloids on viral titers. The effects of alkaloids on virus titers compared to the model group were shown through a forest plot (effect size and 95% confidence interval). Black diamonds represented the pooled effect size. Green squares represent the SMD of each study, with their size indicating the study’s weight. The references cited in 2.1.1 PR8 were [28,29,30,32,33,34]; The reference cited in 2.1.2 FM1 was [26]; The reference cited in 2.1.3 Other was [35].

The effects of alkaloids on viral titers are shown in Figure 5. Six studies contributed to the PR8 subgroup. The pooled SMD was −4.36 [95% CI: −7.57, −1.15], indicating a significant reduction in viral load in the experimental group compared to the control group (Z = 2.67, *p* = 0.008). High heterogeneity was observed (I^2^ = 93%, *p* < 0.00001). For the FM1 subgroup, one study was included, with an SMD of −16.29 [95% CI: −21.39, −11.18], showing a substantial reduction in viral load in the experimental group (Z = 6.26, *p* < 0.00001). No heterogeneity was reported. For other studies, one study was included, with an SMD of 1.38 [95% CI: 0.81, 1.95], suggesting a significant increase in viral load in the experimental group (Z = 4.78, *p* < 0.00001). No heterogeneity was reported. Across all included studies (*n* = 8), the pooled SMD was −4.41 [95% CI: −6.82, −2.00], indicating a significant overall reduction in viral load in the experimental group compared to the control group (Z = 3.59, *p* = 0.0003). Substantial heterogeneity was observed (I^2^ = 95%, *p* < 0.00001).

### 3.6. Effect of Alkaloids on Pulmonary Inflammation Scores

**Figure 6:**
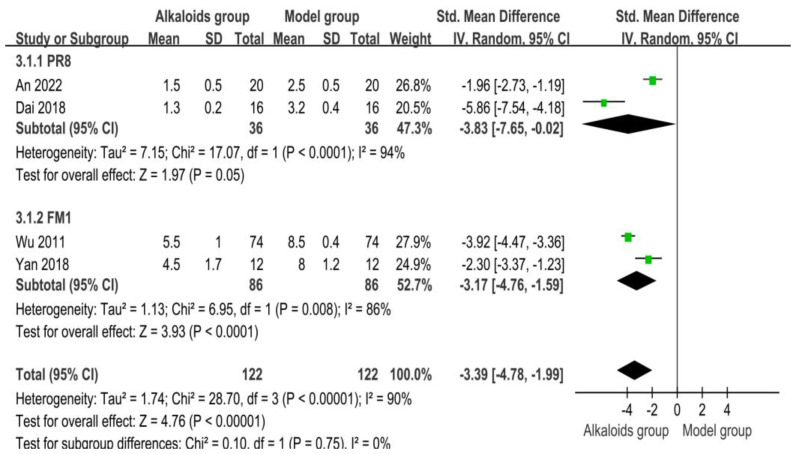
Effects of alkaloids on pulmonary inflammation scores. The effects of alkaloids on pulmonary inflammation scores compared to the model group were shown through a forest plot (effect size and 95% confidence interval). Black diamonds represented the pooled effect size. Green squares represent the SMD of each study, with their size indicating the study’s weight. The references cited in 3.1.1 PR8 were [27,30]; The references cited in 3.1.2 FM1 were [26,31].

The effects of alkaloids on pulmonary inflammation scores are shown in Figure 6. Two studies were included in the PR8 subgroup. The combined SMD was −3.83 (95% CI: −7.65 to −0.02; *p* = 0.05), indicating a significant reduction in pulmonary inflammation scores in the experimental group compared to the control group. However, high heterogeneity was observed (I^2^ = 94%, *p* < 0.0001), suggesting variability among the included studies. Two studies were also included in the FM1 subgroup. The pooled SMD was −3.17 (95% CI: −4.76 to −1.59; *p* < 0.0001), showing a significant reduction in pulmonary inflammation scores for the experimental group. Moderate heterogeneity was detected (I^2^ = 86%, *p* = 0.008), suggesting some level of inconsistency among these studies. When combining all four studies, the overall SMD was −3.39 (95% CI: −4.78 to −1.99; *p* < 0.0001), indicating a significant reduction in pulmonary inflammation scores in the experimental groups across all included studies. High heterogeneity was observed (I^2^ = 90%, *p* < 0.0001). The test for subgroup differences (Chi^2^ = 0.10, df = 1, *p* = 0.75) revealed no significant differences between the PR8 and FM1 subgroups.

### 3.7. Effect of Alkaloids on Lung Index

**Figure 7:**
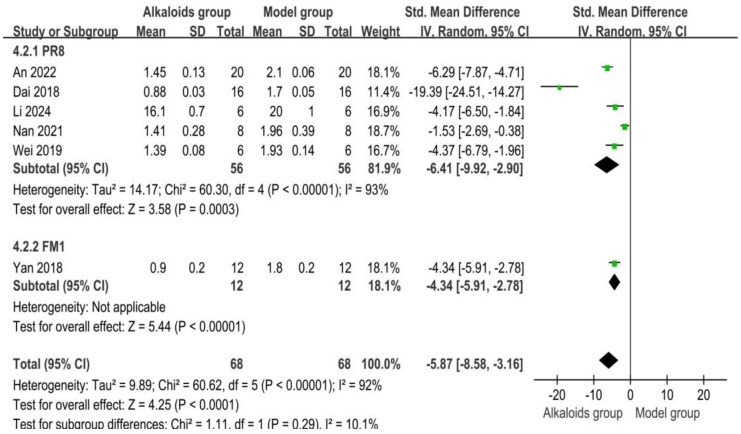
Effects of alkaloids on lung index. The effects of alkaloids on lung index compared to the model group were shown through a forest plot (effect size and 95% confidence interval). Black diamonds represented the pooled effect size. Green squares represent the SMD of each study, with their size indicating the study’s weight. The references cited in 4.2.1 PR8 were [27,29,30,34,36]; The reference cited in 4.2.2 FM1 was [26].

The effects of alkaloids on lung index are shown in Figure 7. Five studies contributed to the PR8 subgroup. The pooled standardized mean difference (SMD) was −6.41 [95% CI: −9.92, −2.90], indicating a significant reduction in lung index in the experimental group compared to the control group (Z = 3.58, *p* = 0.0003). High heterogeneity was observed (I^2^ = 93%, *p* < 0.00001). One study was included in the FM1 subgroup, with an SMD of −4.34 [95% CI: −5.91, −2.78], showing a significant reduction in lung index in the experimental group (Z = 5.44, *p* < 0.00001). No heterogeneity was reported. Across all included studies (*n* = 6), the pooled SMD was −5.87 [95% CI: −8.58, −3.16], indicating a significant overall reduction in lung index in the experimental group compared to the control group (Z = 4.25, *p* < 0.0001). Subgroup differences were not significant (Chi^2^ = 1.11, df = 1, *p* = 0.29; I^2^ = 10.1%).

### 3.8. Effect of Alkaloids on TNF-α and IL-6

**Figure 8:**
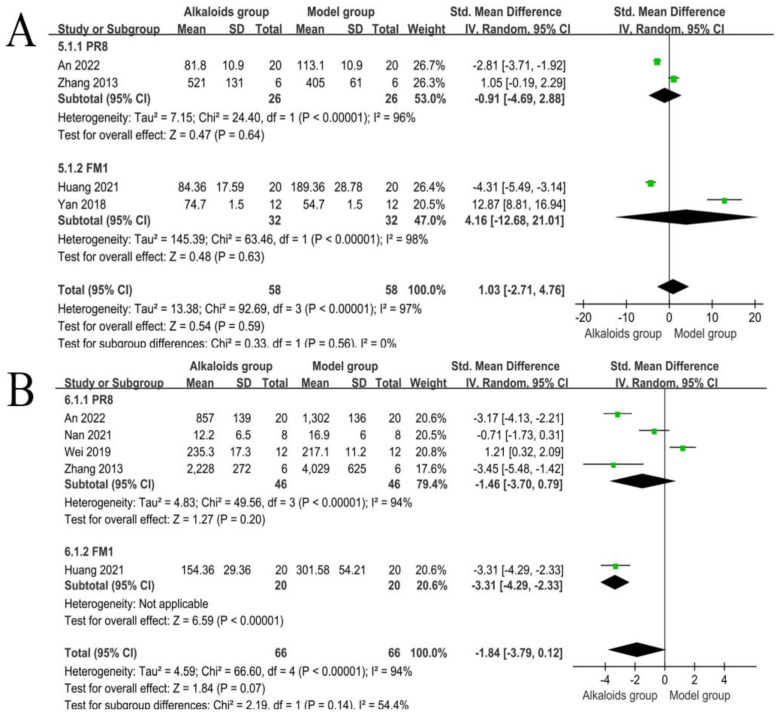
Effects of alkaloids on TNF-α and IL-6. The effects of alkaloids on TNF-α (**A**) and IL-6 (**B**) compared to the model group were shown through a forest plot (effect size and 95% confidence interval). Black diamonds represented the pooled effect size. Green squares represent the SMD of each study, with their size indicating the study’s weight. The references cited in 5.1.1 PR8 were [27,33]; The references cited in 5.1.2 FM1 were [26,37]; The references cited in 6.1.1 PR8 were [27,33,34,36]; The reference cited in 6.1.2 FM1 was [37].

The effects of alkaloids on TNF-α are shown in Figure 8A. For TNF-α, two studies were analyzed in the PR8 subgroup. The combined SMD for this subgroup was −0.91 (95% CI: −4.69 to 2.88; *p* = 0.64), indicating no statistically significant difference between the experimental and control groups. Individual study results varied significantly, with An et al. (2022) reporting an SMD of −2.81 (95% CI: −3.71 to −1.92), while Zhang et al. (2013) showed a positive SMD of 1.05 (95% CI: −0.19 to 2.29). High heterogeneity was observed (I^2^ = 96%, *p* < 0.00001), suggesting substantial variability across the studies. Two studies contributed to the FM1 subgroup. The pooled SMD for this subgroup was 4.16 (95% CI: −12.68 to 21.01; *p* = 0.63), reflecting wide confidence intervals and a lack of statistical significance. Heterogeneity within this subgroup was extremely high (I^2^ = 98%, *p* < 0.00001), indicating inconsistencies in the results. Huang et al. (2021) reported an SMD of −4.31 (95% CI: −5.49 to −3.14), while Yan et al. (2018) demonstrated a highly positive SMD of 12.87 (95% CI: 8.81 to 16.94). Across all four studies, the combined SMD was 1.03 (95% CI: −2.71 to 4.76; *p* = 0.59). The overall analysis suggests no significant difference in TNF-α levels between experimental and control groups. Substantial heterogeneity was noted (I^2^ = 97%, *p* < 0.00001), indicating a high degree of variability among studies. The test for subgroup differences (Chi^2^ = 0.33, *p* = 0.56) showed no significant differences between the PR8 and FM1 subgroups.

The effects of alkaloids on IL-6 are shown in Figure 8B. Four studies contributed to the PR8 subgroup analysis. The pooled SMD for this subgroup was −1.46 (95% CI: −3.70 to 0.79; *p* = 0.20), indicating no statistically significant difference between the experimental and control groups. Notable heterogeneity was observed within this subgroup (I^2^ = 94%, *p* < 0.00001). Individual study results varied, with An et al. (2022) and Zhang et al. (2013) showing significant reductions in IL-6 levels (−3.17 and −3.45, respectively), while Wei et al. (2019) reported a positive SMD (1.21, 95% CI: 0.32 to 2.09). One study was included in the FM1 subgroup. The SMD for this study was −3.31 (95% CI: −4.29 to −2.33; *p* < 0.00001), demonstrating a significant reduction in IL-6 levels in the experimental group. Heterogeneity for this subgroup was not applicable, as only one study was analyzed. The overall pooled analysis across all five studies yielded an SMD of −1.84 (95% CI: −3.79 to 0.12; *p* = 0.07), suggesting a trend toward reduced IL-6 levels in the experimental group, though the result did not reach statistical significance. High heterogeneity was detected (I^2^ = 94%, *p* < 0.00001), reflecting substantial variability between studies. The test for subgroup differences (Chi^2^ = 2.19, *p* = 0.14) revealed no statistically significant differences between the PR8 and FM1 subgroups (I^2^ = 54.4%).

### 3.9. Effect of Alkaloids on Body Weight

**Figure 9:**
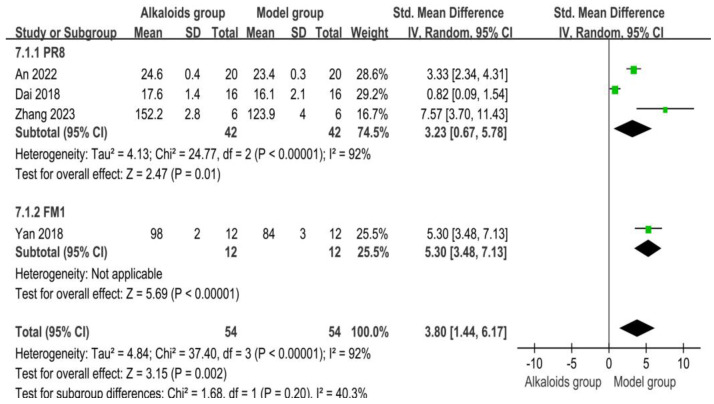
Effects of alkaloids on body weight. The effects of alkaloids on body weight compared to the model group were shown through a forest plot (effect size and 95% confidence interval). Black diamonds represented the pooled effect size. Green squares represent the SMD of each study, with their size indicating the study’s weight. The references cited in 7.1.1 PR8 were [27,30,32]; The reference cited in 7.1.2 FM1 was [26].

The effects of alkaloids on body weight are shown in Figure 9. Three studies were included in the PR8 subgroup analysis. The pooled SMD was 3.23 (95% CI: 0.67 to 5.78; *p* = 0.01), indicating a statistically significant increase in body weight in the experimental group compared to the control group. The individual study results varied, with SMDs ranging from 0.82 (Dai et al. (2018)) to 7.57 (Zhang et al. (2023)). The heterogeneity was substantial (I^2^ = 92%, *p* < 0.00001), suggesting considerable variability among the studies. One study contributed to the FM1 subgroup, with an SMD of 5.30 (95% CI: 3.48 to 7.13; *p* < 0.00001), indicating a significant increase in body weight in the experimental group. Heterogeneity was not applicable as only one study was analyzed. The combined analysis across all four studies yielded an SMD of 3.80 (95% CI: 1.44 to 6.17; *p* = 0.002), suggesting a statistically significant overall increase in body weight for the experimental group. High heterogeneity was observed across all studies (I^2^ = 92%, *p* < 0.00001), reflecting substantial variability between studies. The test for subgroup differences (Chi^2^ = 1.68, *p* = 0.20) indicated no statistically significant differences between the PR8 and FM1 subgroups (I^2^ = 40.3%).

## 4. Discussion

### 4.1. Overview of Effectiveness and Summary of Evidence

There is evidence to suggest that alkaloids play a beneficial role in treating influenza. By collecting and analyzing data from 13 preclinical studies, this systematic review and meta-analysis evaluated the efficacy of alkaloids and their derivatives in treating influenza. The results indicated that alkaloids could improve the primary outcome measures of influenza models, such as viral titers, pulmonary inflammation scores, and survival rates, as well as secondary outcome measures, including lung index and body weight. For pro-inflammatory cytokines, due to insufficient evidence, alkaloid compounds may not effectively reduce the levels of TNF-α and IL-6. The current evidence supports the anti-influenza and anti-inflammatory properties of alkaloids, but their relationship with inflammatory mediators such as IL-6 and TNF-α needs further clarification.

### 4.2. Mechanisms of Alkaloids in Treating Influenza

**Figure 10:**
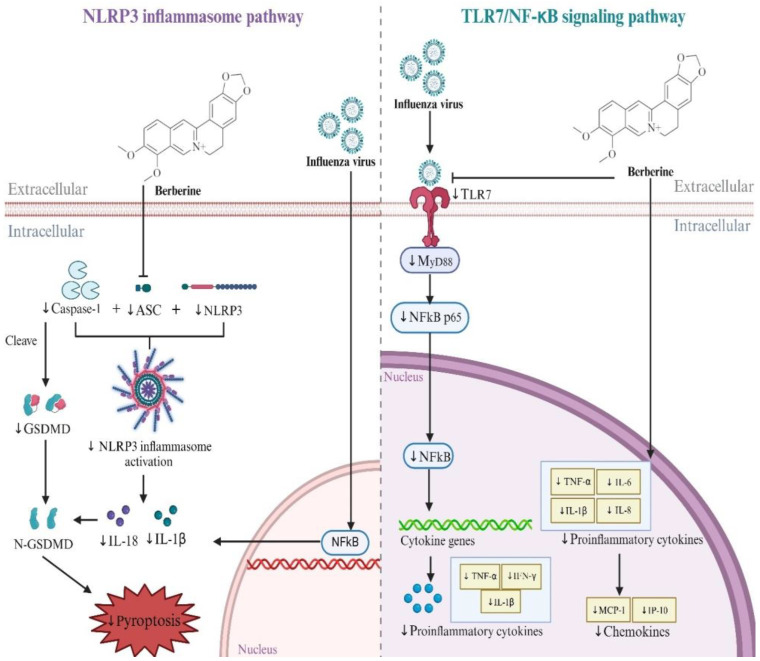
Effect of BBR on NLRP3 inflammasome pathway and TLR7/NF-κB pathway. The downward arrow represented descent (arrows indicating inhibition end with short vertical line).

Multiple studies have confirmed that berberine (BBR) is antiviral, anti-inflammation, and anti-influenza and improves lung pathological change effects against the influenza virus both in vitro and in vivo [31]. The specific mechanism of BBR in treating influenza through the NLRP3 inflammasome pathway and TLR7/NF-κB signaling pathway is shown in Figure 10 and Table 2. Nuclear factor (NF)-κB has been identified as a key promoter for influenza-associated inflammation and effective viral replication [39]. In the NF-κB pathway, Toll-like receptor 7 (TLR7) can recognize viral single-stranded RNA and stimulate down-stream myeloid differentiation factor 88 (MyD88) to activate NF-κB thereby driving adhesion molecules and pro-inflammatory mediators [40,41]. Yan et al. confirmed that BBR strongly inhibits influenza virus replication in A549 cells and mouse lungs. BBR inhibited the up-regulation of TLR7, MyD88, and NF-κB (p65) at the mRNA and protein levels, thereby regulating the TLR7 signaling pathway [26]. Influenza virus infection triggers NLRP3 inflammasome activation to induce pro-inflammatory response and pyroptosis [42,43]. BBR has been shown to improve lung inflammation in influenza mice by inhibiting NACHT, LRR, and PYD domains-containing protein 3 (NLRP3) inflammasome activation and inhibiting GSDMD-mediated apoptosis by reducing GSDMD expression and suppressing NLRP3 inflammasome-mediated GSDMD activation. Specifically, BBR reduced the expression of NLRP3, apoptosis-associated speck-like protein containing a CARD (ASC), and cysteinyl aspartate-specific proteinase (Caspase) 1, and the ratio of Caspase1p20 subunit to Caspase1, thereby inhibiting the activation of NLRP3 inflammasome and leading to a decrease in the levels of mature IL-1β and IL-18 in the lungs [27].

**Figure 11:**
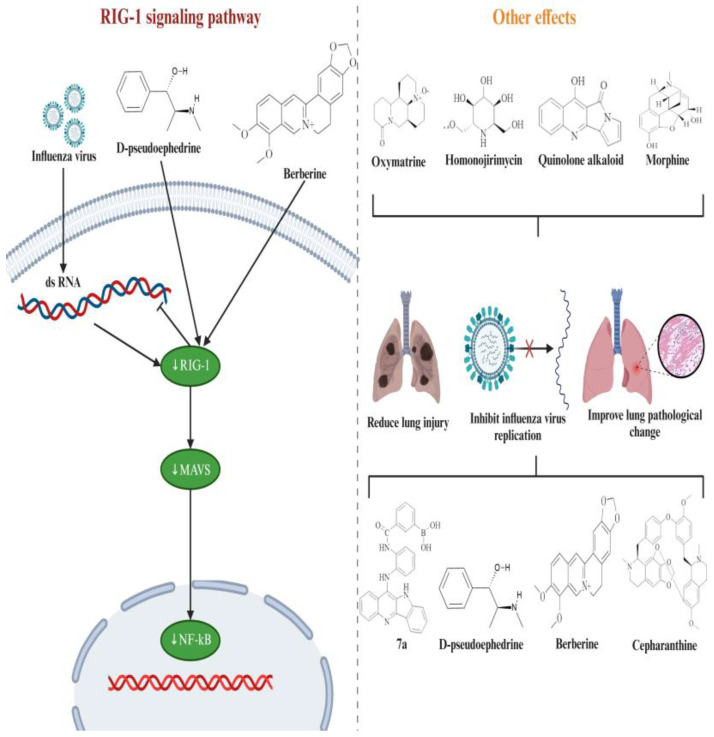
Effect of BBR on RIG-I signaling pathway and other mechanisms of alkaloids in treating influenza. The red X represented the inhibitory effect on the replication of influenza virus.

The other mechanisms of alkaloids in treating influenza are shown in Figure 11 and Table 2. Antiviral innate immune response receptor RIG-I (RIG-I) is a member of the RLR family and can induce innate immunity, inflammation, and gene expressions by detecting viral RNA ligands or processed self RNA in the cytoplasm, thereby limiting infection [44]. It works as the primary receptor to recognize intracellular ssRNA and transcriptional intermediates of influenza virus in infected host cells [45]. The innate immune response of the influenza virus is strictly dependent on RIG-I. Influenza virus infection can produce multiple RIG-I agonists, with the most critical RIG-I stimulant being the virus genome itself [46]. RIG-I exists both in the cytoplasm and nucleus, and the RIG-I signal plays a crucial role in restricting influenza virus replication [47]. For example, Kandasamy et al. showed delayed clearance of influenza virus after infection with influenza A virus in RIG-I deficient mice [48]. Together, during influenza virus infection, the virus-conserved components of pathogen-associated molecular patterns are recognized by host–pathogen recognition receptors, such as RIG-I, leading to the activation of innate immune signals and finally inducing the production of various cytokines and antiviral molecules, inhibiting influenza virus replication [49,50]. Mechanistically, RIG-I signaling mainly reduces influenza virus infection by generating interferon (IFN) [51]. Type I interferons, namely IFN-alpha and beta IFN-α and IFN-β, can prevent virus replication in the host [52]. L-Methylephedrine, L-ephedrine, and D-pseudoephedrine induced a significant increase in IFN-β levels [34]. In addition, in vitro experiments have shown that homoharingtonin can inhibit STING-mediated activation of the IFN-β promoter in a dose-dependent manner [53]. However, the effects of most alkaloids on IFN-α and IFN-β are still unclear, and further research can be conducted in the future. D-pseudoephedrine and BBR could significantly inhibit the mRNA expression of RIG-I, alleviate lung inflammation, improve immune function, and have a protective effect on infected mice [34]. Cepharanthine could reduce viral titers and alleviate pathological damage and inflammation in mouse lung tissue caused by the influenza virus [29]. Dai et al. confirmed that oxymatrine could improve the survival rate of influenza-infected mice, reduce lung index, alleviate lung inflammation, reduce lung virus titers, and improve pathological changes in lung tissue [30]. Homonojirimycin had a protective effect against influenza virus infection, significantly increasing the levels of IFN-γ and IL-10 and reducing the levels of TNF-α and IL-6 [33]. Morphine alleviated lung inflammation in influenza rats but hindered virus clearance from the lungs [35]. Two other derivatives of alkaloids (marine-derived quinolone alkaloid and quindoline derivatives) have been found to have anti-influenza activity and alleviate lung injury [28,32].

Directly targeting the virus to suppress virus replication has become an important component of the treatment strategy for influenza. The neuraminidase dominates the surface of viral particles and is responsible for the infectivity of the virus. It plays a role in virus replication by releasing new virus particles from host cells and separating them from the polysaccharide structure containing neuraminic acid on the surface of infected cells [54]. Studies have found that alkaloids can also inhibit cytopathogenic effects and neuraminidase activity in vitro [17]. The active site of viral neuraminidase can be blocked by berberine derivatives, just as it is blocked by the antiviral drug oseltamivir (a well-known neuraminidase inhibitor) [55]. Molecular docking studies used berberine derivatives and neuraminidase of influenza A and B viruses confirmed their inhibitory effect on viral neuraminidase [56]. Quinine could significantly suppress early steps in the replication cycle of the influenza A virus [57]. However, the mechanism by which most alkaloids directly target viruses is still unclear.

### 4.3. Safety and Bioavailability of Alkaloids

BBR is a famous natural monomer compound that can be easily obtained from traditional medicinal plants or through total synthesis, with a molecular formula of C_20_H_18_NO_4_^+^ and a molecular weight of 336.37 g/mol [58]. BBR is a yellow crystalline powder, odorless, with characteristic alkaloid bitterness [59]. Free BBR can slowly dissolve in water and is easily soluble in hot ethanol. Its hydrochloride is not easily soluble in water and is more soluble in boiling water, while its sulfate is relatively water-soluble [60]. In recent years, significant progress has been made in the extraction methods of BBR. Chloride or sulfate of BBR is used as raw material for preparing oral tablets or capsules for clinical purposes [61]. Research has confirmed that BBR is almost safe at conventional doses, with a relatively low incidence of adverse reactions such as gastrointestinal discomfort and transient increases in plasma bilirubin levels [62]. However, due to poor absorption from the gut, liver first-pass elimination, and rapid metabolism in the body, the oral bioavailability of BBR is low, which limits its application [63]. Hence, various strategies have been developed to enhance the bioavailability and pharmacological activity of berberine, including using P-glycoprotein inhibitors as adjuvants for BBR, using penetration enhancers, and constructing lipid particle delivery systems [64]. In addition, structural modification of BBR can also improve its bioavailability. Dihydroberberine and 8,8-dimethyldihydroberberine have better bioavailability [60]. After oral administration, the levels of BBR and its bioactive metabolites in organs are higher than those in the blood. BBR has a fast organ distribution, including liver, kidney, muscle, lung, brain, heart, pancreas, and fat, and remains generally stable for 48 h [65,66].

D-pseudoephedrine is commonly used for symptomatic treatment of common cold, sinusitis, and influenza [67]. D-pseudoephedrine is easily absorbed from the gastrointestinal tract and takes effect after 30 min oral administration, reaching its maximum concentration 1–4 h later. It is mainly excreted unchanged through urine. Adverse reactions of D-pseudoephedrine may occur during oral and intranasal administration after a single dose or after prolonged (5 days) treatment, including central nervous system stimulation (incidence > 30%), digestive tract dysfunction (incidence > 5%), allergic reactions, psychological dependence, cardiac arrhythmias, and elevated blood pressure [68,69].

Cepharantine is an active ingredient isolated and extracted from *Stephania cepharantha Hayata*, which can be used clinically in both oral and injectable administration forms such as oral tablets and powders [70]. A daily dose of 1–60 mg has been safely and effectively used to treat various diseases [71]. Since 1950, no safety issues have been observed, and no serious side effects have been found in the clinical use of cepharantine [72]. Its poor water solubility leads to low oral bioavailability, and after absorption, it is mainly distributed throughout the liver, kidneys, spleen, and lungs [73].

Oxymatrine can be extracted from *Sophora flavescens* and converted into matrine in the body [74]. It is one of the typical alkaloids containing a quinolizidine backbone. Due to its low membrane permeability, greater hepatic clearance, and biotransformation in the gastrointestinal tract, oral bioavailability is low. The time for the drug concentration to reach its peak is 1.58 h, with a half-life of 3.44 h [75]. At high doses, OMT will become a toxic substance to the liver [76]. *Commelina communis* L., also known as dayflower, is traditionally used in China to treat non-infectious fever. Homonojirimycin is one of the alkaloids, with little research on its bioavailability and safety.

### 4.4. Future Prospects on Alkaloids

In conclusion, BBR has a clear metabolic pathway, low potential toxicity, and significant therapeutic effects, making it the most promising potential new drug for treating influenza [77]. In addition, multiple studies have attempted to address the issue of low bioavailability. However, there are still some gaps and limitations in current research. First, the molecular mechanism of BBR in human subjects has not been completely revealed; the specific targets and mechanisms in humans are still unclear. Secondly, improving the bioavailability of BBR is also one of the issues that need to be addressed in the future, and developing modification strategies that can be practically applied is crucial. In the future, more in vitro and in vivo studies are needed to elucidate the molecular mechanisms of BBR therapy for influenza. It is necessary to conduct well-designed, large-scale, long-term, and high-quality multicenter clinical trials to evaluate the effectiveness and safety of BBR in treating influenza, as well as to promote its development and clinical application.

Presently, the commercial preparations of cepharanthine are ordinary tablets, and several new dosages (such as oral disintegrating tablets and nanoformulations) have limitations to use in actual clinical practice. Although cephalanthine has been clinically used for a long time and has strong safety, its detailed mechanism for treating influenza is still unclear due to limited research. Further research is urgently needed on in vivo models and clinical trials to confirm the role of cepharantine in real-life influenza cases and to fully unleash the potential of this natural alkaloid. The research on the treatment of influenza with D-pseudoephedrine and cephalanthine is similar, but the safety of D-pseudoephedrine is worse. There has not yet been a technologically advanced method to extract a large amount of oxymatrine, homonojirimycin, 7a, and quinolone alkaloid, and it is necessary to conduct in-depth research on them to provide sufficient human safety and achieve therapeutic benefits for influenza.

### 4.5. Advantages and Limitations of This Study

The key advantages of our study are as follows: (i) We systematically evaluated and analyzed the real effects and limitations of alkaloids in treating influenza through animal experiments and pointed out the current research problems and improvement directions. (ii) As the first study in the current field, we integrated multiple outcome measures to comprehensively reveal the roles of various alkaloids throughout the entire treatment process. It has significant value for designing relevant future animal and clinical research. (iii) We conducted a strict assessment of the internal bias risk in animal experiments based on the internationally recognized SYRCLE bias risk assessment tool. The internal and external validity of the current animal experiments were explored. Then, we pointed out the problems in the design and implementation of animal research in this field and proposed suggestions to improve its quality.

However, this systematic review and meta-analysis should consider several limitations as follows: (i) Since the number of studies on a single type of alkaloid for influenza treatment is insufficient to support a meta-analysis, we combined the data of all alkaloid monomers for analysis to confirm that alkaloids can effectively treat influenza. The combined data analysis of all alkaloid monomers may ignore individual differences and cannot accurately reflect the specific efficacy of each monomer. We will continue to pay attention to this field in the future and wait for further analysis when there is enough research on monomer alkaloids. (ii) The execution process of preclinical research is very complex, with multiple interfering factors leading to the introduction of bias. This complexity may be a key factor leading to high heterogeneity between studies. Due to the vague identification of the sources of heterogeneity, we conducted a meta-analysis using a random-effects model, which resulted in our conclusions being more conservative. (iii) While the results demonstrate promising effects of alkaloids in animal models, their applicability to human clinical settings remains uncertain and requires further validation.

## 5. Conclusions

Alkaloids can reduce viral load in the lungs, have a protective effect on lung function, alleviate pathological changes, improve survival rates, and increase body weight. However, analysis shows that alkaloids have no effect on IL-6 and TNF-α levels due to the methodological quality and heterogeneity of the study. The anti-influenza and anti-inflammatory properties of alkaloids may involve the NLRP3 inflammasome pathway, TLR/NF-κB signaling pathway, and RIG-I pathway. This study provides evidence supporting alkaloids as potential candidates for treating influenza. However, to accurately evaluate the anti-influenza properties of alkaloids and provide higher levels of evidence before clinical application, more extensive, longer, and higher-quality preclinical studies are needed. Meanwhile, future research needs to further standardize the implementation and reporting of animal experiments to improve the quality of evidence in preclinical studies and reduce the risk of translating preclinical research results into clinical practice.

## Figures and Tables

**Table 1:** Basic characteristics of the included studies.

Study	Species (Strain; Age; Weight)	Sample Size (T/M)	Modeling Method	Intervention			Outcomes
				Nature/Subclass	Administration	Dose/Duration	
[26]	Mice (C57BL/6; 6–8 weeks; 18–22 g)	12/12	A/FM1/1/47 (H1N1) influenza in suspension infected intranasally (50 µL 5 × 10^3^)	Berberine/Isoquinoline	Oral gavage	20 mg/kg/7 days	1. Viral titer; 2. Pulmonary inflammation scores; 3. Lung index; 4. TNF-α and IL-1β; 5. Body weight.
[27]	Mice (BALB/c; 6–7 weeks; 14–16 g)	20/20	Sterile PBS containing A/Puerto Rico/8/34 (H1N1) intranasally inoculated 25 μL 10LD_50_ (LD_50_: 10^−5.82^)	Berberine/Isoquinoline	Intraperitoneal injection	10 mg/kg/7 days	1. Survival rate; 2. Pulmonary inflammation scores; 3. Lung index; 4. TNF-α and IL-6; 5. Body weight.
[28]	Mice (Kunming; 4 weeks; 12–16 g)	/	Influenza virus (A/Puerto Rico/8/34 (4HAU/mouse) [H1N1] diluted in 40 µL of 1 × PBS inoculated intranasal	7a/Isoquinoline	Oral gavage	10 mg/kg/4 days	1. Viral titer; 2. Survival rate.
[29]	Mice (BALB/c; 4–5 weeks; 16–18 g)	6/6	Influenza virus A/PR/8/34 (PR8) (H1N1) 50 µL 5LD_50_ intranasally infected	Cepharanthine/Isoquinoline	Oral gavage	60 mg/kg/5 days	1. Viral titer; 2. Lung index.
[30]	Mice (C57BL/6J; 6–8 weeks; 18–22 g)	16/16	Infect intranasally with 10 × MLD_50_ of influenza A virus (PR8) viruses in a 50 μL volumes	Oxymatrine/Isoquinoline	Oral gavage	120 mg/kg/6 days	1. Viral titer; 2. Pulmonary inflammation scores; 3. Lung index.
[31]	Mice (BALB/c; 4–5 weeks; 16–18 g)	54/54	Influenza virus A/FM/1/47 (H1N1) intranasally infect with 50% LD_50_ influenza virus in a 25 μL volume	Berberine/Isoquinoline	Intraperitoneal injection	0.005 g/kg/7 days	1. Pulmonary inflammation scores.
[32]	Mice (BALB/c; 4 weeks; 12–16 g)	/	Influenza virus (A/Puerto Rico/8/34 [H1N1]; PR/8) inoculate intranasally with 500 PFU/mouse in 40 μL of 1 × PBS	Quinolone alkaloid/Quinoline	Oral gavage	2.5 mg/kg/4 days	1. Viral titer.
[33]	Mice (BALB/c; 18–22 g)	/	Influenza A virus (A/PR/8/34 H1N1, PR8) infect intranasally with 5 × ID_50_ suspension in PBS	Homonojirimycin	Oral gavage	1 mg/kg/8 days	1. Viral titer; 2. Survival rate; 3. TNF-α and IL-6.
[34]	Mice (ICR; 18–22 g)	12/12	Mouse-adapted influenza virus A/PR8/34 (H1N1) infect intranasally with 10LD_50_ in 50 μL PBS	D-pseudoephedrine/Amine	Oral gavage	20 mg/kg/7 days	1. Viral titer; 2. Lung index; 3. IL-1β and IL-6.
[35]	Rats (Lewis)	/	Infect 2.0 × 10^4^ PFU of rat-adapted influenza virus	Morphine/Isoquinoline	Implant subcutaneously	75 mg	1. Viral titer.
[36]	Mice (BALB/c; 13–15 g)	8/8	PR8 (H1N1) intranasally inoculated 25 μL 10LD_50_ (LD_50_: 10^−2.68^)	Berberine/Isoquinoline	intraperitoneal injection	5 mg/kg/6 days	1. Lung index; 2. Body weight; 3. IL-6.
[37]	Mice (3 weeks)	20/20	FM1 (H1N1) intranasally inoculated 50 μL LD_50_ (LD_50_: 2^−2.83^/50 μL)	Berberine/Isoquinoline	Oral gavage	1 mg/mL/5 days	1. TNF-α and IL-6.
[38]	Mice (C57BL/6; 8 weeks)	10/10	FM1 (H1N1) intranasally inoculated 50 μL LD_50_ (LD_50_: 2^−2.83^/50 μL)	Berberine/Isoquinoline	Oral gavage	0.02 g/kg/5 days	1. Survival rate.

Abbreviations: Median lethal dose (LD_50_), median infectious dose (ID_50_).

**Table 2:** Mechanisms of alkaloids in treating influenza.

Study	Mechanisms
[26]	1. Relieving pulmonary inflammation and necrosis, inflammatory cell infiltration, and pulmonary edema; 2. Inhibiting TLR7/NF-κB signaling pathway (decrease TLR7, MyD88, and NF-κB (p65) at both the mRNA and protein levels); 3. Inhibiting influenza virus replication, T cell responses, and production of inflammatory cytokines.
[27]	1. Protecting mice from H1N1 challenge; 2. Alleviating pulmonary inflammation; 3. Inhibiting NLRP3 inflammasome activation (decrease NLRP3, ASC, and Caspase1 at both the mRNA and protein levels, decrease Caspase1p20 subunit/Caspase1 ratio) to ameliorate lung inflammation (decrease IL-1β and IL-18); 4. Inhibiting GSDMD/NLRP3 inflammasome to suppress pyroptosis (inhibit the GSDMD expression and GSDMD activation).
[28]	1. Inhibiting influenza virus multiplication; 2. Reducing lung injury.
[29]	1. Alleviating inflammation and injury in pulmonary tissue; 2. Inhibiting the mRNA expression of the cytokines and chemokines (IL-1β, IL-6, TNF-α, IL-8, MCP-1, and IP-10).
[30]	1. Ameliorating lung inflammation (reduce the transcriptions of IL-6, TNF-α, and IL-1β); 2. Reducing lung injury; 3. Improving lung pathological changes.
[31]	1. Reducing virus titters; 2. Improving lung pathological changes; 3. Inhibiting NO and iNOS production in the lungs; 4. Repressing TNF-α and MCP-1 transcription and expression.
[32]	1. Possessing anti-influenza activities; 2. Improving lung pathological changes.
[33]	1. Reducing virus yields in lungs; 2. Increasing IFN-γ and IL-10; 3. Decreasing TNF-α and IL-6.
[34]	1. Improving lung pathological changes; 2. Reducing virus load in the lung; 3. Relieving pulmonary inflammation (inhibit IL-1β, increase IL-6, and accelerate IL-10) 4. Inhibiting TNF-α mRNA and promoting IFN-γ mRNA; 5. Adjusting the TLRs and RIG-I pathways (down-regulate protein expression levels of MyD88, NF-κB p65, TLR4, and TLR7.
[35]	1. alleviating lung inflammation;
[36]	1. Improving lung pathological changes; 2. Inhibiting mRNA transcription level of IFN-γ, IL-10, and CCL25; 3. Inhibiting IFN-γ.
[37]	1. Improving lung pathological changes; 2. Adjusting the proportion of T lymphocyte subsets; 3. Inhibiting protein level of IL-6, TNF-α, RIG-I, MAVS, and NF-κB; 4. Increase IL-4 level.
[38]	1. Improving lung pathological changes; 2. Down-regulating protein and mRNA expression levels RIG-I, MAVS, and NF-κB in the RLH signaling pathway.

Abbreviations: Tumor necrosis factor (TNF), interferon-γ (IFN-γ), interleukin-6 (IL-6), interleukin-10 (IL-10), myeloid differentiation factor 88 (MyD88), Toll-like receptor (TLR).
